# Supplementary material for: Ontology-driven weak supervision for clinical entity classification in electronic health records
Source: Nat Commun. 2021 Apr 1;12:2017. doi: 10.1038/s41467-021-22328-4 (PMC8016863; doi:10.1038/s41467-021-22328-4)
Supplement: Supplementary file 2 — Reporting Summary [file 41467_2021_22328_MOESM2_ESM.pdf]

nature research

Corresponding author(s): Jason Alan Fries

Last updated by author(s): Feb 23, 2021

Reporting Summary

Nature Research wishes to improve the reproducibility of the work that we publish. This form provides structure for consistency and transparency in reporting. For further information on Nature Research policies, see our Editorial Policies and the Editorial Policy Checklist.

Statistics

For all statistical analyses, confirm that the following items are present in the figure legend, table legend, main text, or Methods section.

n/a	Confirmed	
☐	☒	The exact sample size (n) for each experimental group/condition, given as a discrete number and unit of measurement
☐	☒	A statement on whether measurements were taken from distinct samples or whether the same sample was measured repeatedly
☐	☒	The statistical test(s) used AND whether they are one- or two-sided<br>*Only common tests should be described solely by name; describe more complex techniques in the Methods section.*
☒	☐	A description of all covariates tested
☒	☐	A description of any assumptions or corrections, such as tests of normality and adjustment for multiple comparisons
☐	☒	A full description of the statistical parameters including central tendency (e.g. means) or other basic estimates (e.g. regression coefficient) AND variation (e.g. standard deviation) or associated estimates of uncertainty (e.g. confidence intervals)
☐	☒	For null hypothesis testing, the test statistic (e.g. F, t, r) with confidence intervals, effect sizes, degrees of freedom and P value noted<br>*Give P values as exact values whenever suitable.*
☒	☐	For Bayesian analysis, information on the choice of priors and Markov chain Monte Carlo settings
☒	☐	For hierarchical and complex designs, identification of the appropriate level for tests and full reporting of outcomes
☒	☐	Estimates of effect sizes (e.g. Cohen's d, Pearson's r), indicating how they were calculated

Our web collection on statistics for biologists contains articles on many of the points above.

Software and code

Policy information about availability of computer code

Data collection	No software was used.
Data analysis	Trove is written in Python v3.6, spaCy 2.3.4 was used for NLP preprocessing, and Snorkel v0.9.5 was used for training the label model. BioBERT-Base v1.1, Transformers v2.8, and PyTorch v1.1.0 were used to train all discriminative models. Trove is open source software and publicly available at https://github.com/som-shahlab/trove

For manuscripts utilizing custom algorithms or software that are central to the research but not yet described in published literature, software must be made available to editors and reviewers. We strongly encourage code deposition in a community repository (e.g. GitHub). See the Nature Research guidelines for submitting code & software for further information.

Data

Policy information about availability of data

All manuscripts must include a data availability statement. This statement should provide the following information, where applicable:
- Accession codes, unique identifiers, or web links for publicly available datasets
- A list of figures that have associated raw data
- A description of any restrictions on data availability

All primary data that support the findings of this study are available via public benchmark datasets (BC5CDR, https://biocreative.bioinformatics.udel.edu/tasks/biocreative-v/track-3-cdr/) or are otherwise available per data use agreements with the respective data owners (ShARe/CLEF 2014, https://physionet.org/content/shareclefehealth2014task2/1.0/; THYME, https://healthnlp.hms.harvard.edu/center/pages/data-sets.html; i2b2/n2c2 2009, https://portal.dbmi.harvard.edu/projects/n2c2-nlp/). The data that support the findings of the clinical case study are available on request from the corresponding author JF. These data are not publicly available because they contain information that could compromise patient privacy.

Trove requires access to the UMLS, which is available by license from National Library of Medicine, Department of Health and Human Services, https://www.nlm.nih.gov/research/umls/index.html. Open source ontologies used in this study are available at: SPECIALIST Lexicon, https://lsg3.nlm.nih.gov/LexSysGroup/Summary/lexicon.html; Disease Ontology, https://bioportal.bioontology.org/ontologies/DOID; Chemical Entities of Biological Interest (ChEBI), ftp://ftp.ebi.ac.uk/pub/databases/chebi/; Comparative Toxicogenomics Database (CTD), http://ctdbase.org; AutoNER core dictionary, https://github.com/shangjingbo1226/AutoNER/blob/master/data/BC5CDR/dict_core.txt; ADAM abbreviations database, http://arrowsmith.psych.uic.edu/arrowsmith_uic/adam.html; and the Clinical Abbreviation Recognition and Disambiguation (CARD) framework, https://sbmi.uth.edu/ccb/resources/abbreviation.htm.

Field-specific reporting

Please select the one below that is the best fit for your research. If you are not sure, read the appropriate sections before making your selection.

☒ Life sciences ☐ Behavioural & social sciences ☐ Ecological, evolutionary & environmental sciences

For a reference copy of the document with all sections, see nature.com/documents/nr-reporting-summary-flat.pdf

Life sciences study design

All studies must disclose on these points even when the disclosure is negative.

Sample size	Sample sizes for our all our six entity benchmarking were dictated by the original 3rd party dataset creators, with entity sample sizes of n=2819, n=4424, n=5385, n=7367, n=7367, and n=18990. These standard benchmark datasets reflect a wide range of clinical NLP tasks and are annotated and validated using standard practices by the larger community of clinical NLP researchers.
Data exclusions	No exclusions.
Replication	All NLP models were verified and replicated using standard machine learning practices, including a held out (unseen) test set for computing performance metrics and running n=10 replicates for each model training process using different random seeds in order to measure the variance of trained models.
Randomization	For entity benchmarking, samples were generated randomly by 3rd parties. For our internal Stanford COVID-19 experiments, subjects (i.e., patient notes) were selected uniform at random from our emergency department population.
Blinding	Blinding was not relevant to out study. We do not analyze interventions or other allocations across different groups, but instead characterize methods for analyzing textual documents that were generated by 3rd parties or sampled uniform at random (i.e., agnostic to group attributes) from our Stanford emergency department population.

Reporting for specific materials, systems and methods

We require information from authors about some types of materials, experimental systems and methods used in many studies. Here, indicate whether each material, system or method listed is relevant to your study. If you are not sure if a list item applies to your research, read the appropriate section before selecting a response.

Materials & experimental systems

n/a	Involved in the study	
☒	☐	Antibodies
☒	☐	Eukaryotic cell lines
☒	☐	Palaeontology and archaeology
☒	☐	Animals and other organisms
☐	☒	Human research participants
☒	☐	Clinical data
☒	☐	Dual use research of concern

Methods

n/a	Involved in the study	
☒	☐	ChIP-seq
☒	☐	Flow cytometry
☒	☐	MRI-based neuroimaging

Human research participants

Policy information about studies involving human research participants

Population characteristics	We examine the text data of notes generated by patient visits to the Stanford Health Care (SHC) emergency department for COVID-19 testing. These clinical notes do not contain structured data on age, gender, race/ethnicity, etc. and our analyses do not consider these attributes in our analyses.
Recruitment	There was no recruitment done for this study. Subjects (patient notes) were sampled from the SHC emergency department where notes are generated as part of a patient visit based on the need for a COVID-19 test. Our paper explores NLP methods for machine reading the notes generated during this process, thus the impact of patient self-selection bias should be minimal to non-existent.
Ethics oversight	Our study was approved by the Stanford University Administrative Panel on Human Subjects Research, protocol #24883

Note that full information on the approval of the study protocol must also be provided in the manuscript.
